# The relationship between clinical outcomes and empirical antibiotic therapy in patients with community-onset Gram-negative bloodstream infections: a cohort study from a large teaching hospital

**DOI:** 10.1017/S0950268820002083

**Published:** 2020-09-11

**Authors:** A. Aryee, P. Rockenschaub, M. J. Gill, A. Hayward, L. Shallcross

**Affiliations:** 1 Institute of Health Informatics, University College London, London, United Kingdom of Great Britain and Northern Ireland; 2 Department of Microbiology, Queen Elizabeth Hospital Birmingham, Birmingham, United Kingdom of Great Britain and Northern Ireland; 3 Institute of Epidemiology and Health Care, University College London, London, United Kingdom of Great Britain and Northern Ireland

**Keywords:** Antibiotic resistance, bloodstream infections, *Escherichia coli* (*E. coli*), Gram-negative bacteria, Klebsiella

## Abstract

Antibiotic-resistant Gram-negative bacteraemias (GNB) are increasing in incidence. We aimed to investigate the impact of empirical antibiotic therapy on clinical outcomes by carrying out an observational 6-year cohort study of patients at a teaching hospital with community-onset *Escherichia coli* bacteraemia (ECB), *Klebsiella pneumoniae* bacteraemia (KPB) and *Pseudomonas aeruginosa* bacteraemia (PsAB). Antibiotic therapy was considered concordant if the organism was sensitive in vitro and discordant if resistant. We estimated the association between concordant *vs.* discordant empirical antibiotic therapy on odds of in-hospital death and ICU admission for KPB and ECB. Of 1380 patients, 1103 (79.9%) had ECB, 189 (13.7%) KPB and 88 (6.4%) PsAB. Discordant therapy was not associated with increased odds of either outcome. For ECB, severe illness and non-urinary source were associated with increased odds of both outcomes (OR of in-hospital death for non-urinary source 3.21, 95% CI 1.73–5.97). For KPB, discordant therapy was associated with in-hospital death on univariable but not multivariable analysis. Illness severity was associated with increased odds of both outcomes. These findings suggest broadening of therapy for low-risk patients with community-onset GNB is not warranted. Future research should focus on the relationship between patient outcomes, clinical factors, infection focus and causative organism and resistance profile.

## Introduction

Reducing the rates of Gram-negative bacteraemia (GNB) and antimicrobial resistance (AMR) are public health priorities, with a major focus on reducing antibiotic prescribing given the undeniable link between prescribing and AMR. Despite reductions in total antibiotic consumption, rates of antibiotic-resistant GNB continue to rise due to a year-on-year increase in incidence, as well as increases in the proportions of resistant isolates [1]. Mandatory surveillance of *Escherichia coli* bacteraemia (ECB) in England in 2019 shows that it comprises 50% of all bacteraemias, but 73% of antibiotic-resistant bacteraemias, with approximately 70% being community-onset [1].

The importance of timely broad-spectrum empirical antibiotic therapy in severe infections is emphasised by initiatives such as the Surviving Sepsis campaign, but there is also a focus on reducing broad-spectrum antibiotics as a means of combating AMR [2–4]. Evidence regarding the effect on clinical outcomes of empirical antibiotic therapy to which the bacteraemia organism is resistant in-vitro (discordant antibiotic treatment) is conflicting. Studies examining outcomes in ECB have shown a wide range of case fatality rates (8%–41.5%) and discrepant results on the effect of discordant antibiotic treatment on mortality and length of stay [5–10]. Studies showing an association between discordant antibiotic treatment and increased mortality in GNB have largely been in critical care settings. Therefore, the relationship may be confounded by illness severity and the results may not be generally applied to all patients with community-onset GNB. This notion is supported by findings from a systematic review, which highlighted methodological limitations of studies assessing mortality risk associated with antibiotic treatment in bloodstream infections and the importance of controlling for disease severity [11]. Source of bacteraemia has also been posited as an important predictor of patient outcomes, with several studies finding lower mortality in patients with a urinary compared with non-urinary source ECB [5, 12–14].

To support empirical prescribing decisions, we aimed to investigate the relationship between concordant *vs.* discordant empirical antibiotic treatment on the clinical outcomes of adult patients with community-onset GNB, adjusting for demographic and clinical factors including the severity of illness and source of bacteraemia.

## Methods

This retrospective cohort study used data collected routinely from adult patients admitted to Queen Elizabeth Hospital Birmingham (QEHB) with community-onset bacteraemia due to *Escherichia coli, Klebsiella pneumoniae* and *Pseudomonas aeruginosa*. QEHB, part of University Hospitals Birmingham NHS Foundation Trust, is one of the largest teaching hospital trusts in England, treating approximately 1.3 million people yearly. The trust has well-established electronic healthcare records, including electronic prescribing. In order to include adults with community-onset GNB, patients >18 years of age admitted to QEHB within ±1 day of positive blood culture with the three above mentioned organisms being received in the laboratory during the study period 1 September 2011–1 January 2018 were eligible for inclusion. Patients were included in the study if they had antibiotic prescription data available ±1 day from admission, as this indicated empirical antibiotic treatment and also captured patients treated in the emergency department prior to admission.

For patients with multiple admissions during the study period, we selected only the first admission and excluded subsequent admissions from the analysis and then included only the first blood culture specimen per patient. Patients with polymicrobial bacteraemia were excluded. For specimens with multiple antibiotic phenotypic variants of the same species, the susceptibilities were aggregated and defined as the most resistant phenotype found for that organism. Patients entered the study on the date of admission and exited on the date of death or discharge.

### Data sources

All data were extracted from electronic health records. De-identified data were transferred to the UCL Data Safe haven for secure storage. Microbiology data included all blood cultures positive for the three organisms received in the microbiology laboratory at QEHB during the study period, including antibiotic susceptibilities. Organisms were identified and susceptibility was tested using Vitek 2 (bioMérieux) Advanced Expert System that currently exists, to designate susceptibility categories. Data on positive urine cultures submitted from patients at QEHB, community hospitals Mosely Hall Hospital and West Heath Hospital, and GP surgeries within a date of −30 to +2 days of the admission start date were also extracted in order to identify urinary source bacteraemias. The source was classified as urinary if either the primary or secondary ICD-10 code for the admission indicated this and/or the patient had a positive urine culture where the organism matched that isolated on blood culture (code list and inclusion diagram in Appendix).

**Fig. 1.**
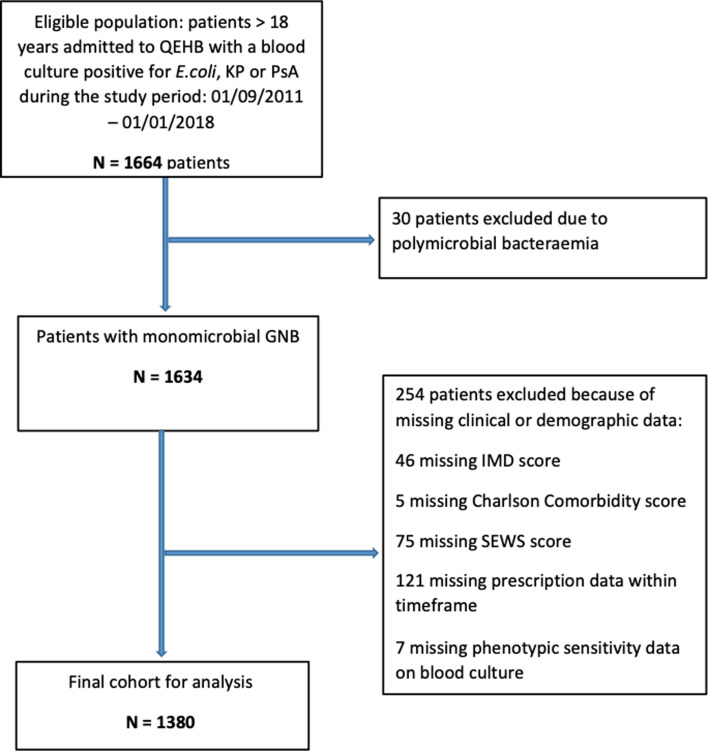
Study inclusion flowchart.

Admission data were extracted from the Patient Administration System (PAS). Index of Multiple Deprivation (IMD) score data, based on patient postcode, was also extracted from PAS, in addition to data on age, sex, ethnicity and ICD-10 codes. Comorbidities were identified through ICD-10 codes and classified using the updated Charlson Comorbidity Index (uCCI) [15, 16]. These were then categorised as a low (uCCI score 0–3) or high (uCCI score ≥4) comorbidity category [17]. Antibiotic prescription data and standardised early warning scores (SEWS, the multi-parameter physiological trigger system used at QEHB during the study period) at admission were extracted from the electronic prescribing system at QEHB. SEWS scores were categorised as low (0–3), mid-level (4–5, the trigger for medical review) and critical (≥6, indicating critical illness) [18]. The study inclusion flowchart is shown in Figure 1.

Empirical antibiotic treatment was considered concordant if the patient was treated intravenously with an antibiotic to which the bacteraemia organism was phenotypically sensitive and discordant if they were treated with an antibiotic to which it was phenotypically resistant or intermediately resistant. Oral/enteral antibiotic treatment was considered discordant even if the organism was phenotypically sensitive, unless the antibiotic prescribed was ciprofloxacin, as a number of studies have found oral therapy to be equivalent to intravenous [19–21].

### Measurement of exposures and covariates

The primary outcome was in-hospital death and the secondary outcome was ICU admission. Covariates were demographic variables (age, sex, ethnicity and IMD score), uCCI score, SEWS score, source of bacteraemia (urinary *vs.* non-urinary) and discordant empirical antibiotic therapy.

### Statistical analysis

A complete case analysis was chosen given the high quality of the data, with <3% missing data across demographic variables. After the initial descriptive analysis of the cohort, we estimated the proportion of patients with each outcome for each of the covariates. For ECB and KPB, we estimated crude associations (odds ratios) between each of the covariates and the outcomes using the Mantel-Haenszel method. A final multivariable-adjusted model was fitted including all predictors with a *P*-value <0.2 in the univariate analysis, in addition to the variables for age (defined as a continuous variable), sex, discordant treatment and urinary source, which were included *a priori*. A formal power calculation was not undertaken, as the study was based on the available population in the dataset. Regression modelling was done using STATA 15.

### Ethics

As this study was a service evaluation, formal ethical approval was not sought. The study was registered as an audit with QEHB in August 2018 (registration number CARMS-13820).

## Results

In total, 1380 patients with bacteraemia were included in the study with a median age of 72 years (IQR 58–83). Of the included patients 1103 had ECB (79.9%), 189 (13.7%) KPB and 88 (6.4%) PsAB, Table 1. A larger proportion of women had ECB (55.7%), but this trend was reversed for KPB and PsAB, where men accounted for 59.8% and 61.4%, respectively. Urinary source bacteraemia was identified in 652 patients (47.3% of patients overall), with proportions varying by organism: 51.4% for ECB, 34.9% for KPB and 21.6% for PsAB.

**Table 1.** Baseline characteristics of patients admitted to QEHB with Gram-negative bacteraemia

Characteristic	All organisms *N* (%)	ECB *N* (%)	KPB *N* (%)	PsAB *N* (%)
Total	1380 (100.0)	1103 (100.0)	189 (100.0)	88 (100.0)
Male gender	656 (47.5)	489 (44.3)	113 (59.8)	54 (61.4)
Urinary source	652 (47.3)	567 (51.4)	66 (34.9)	19 (21.6)
Discordant antibiotic treatment	202 (14.6)	155 (14.1)	23 (12.2)	24 (27.3)
Age group				
18–40	102 (7.4)	79 (7.2)	17 (9.0)	6 (6.8)
41–60	271 (19.6)	202 (18.3)	40 (21.2)	29 (33.0)
61–80	591 (42.8)	459 (41.6)	97 (51.3)	35 (39.8)
>80	416 (30.1)	363 (32.9)	35 (18.5)	18 (20.5)
Ethnicity				
White^a^	1048 (75.9)	843 (76.4)	132 (69.8)	73 (83.0)
Black^b^	62 (4.5)	46 (4.2)	13 (6.9)	3 (3.4)
Asian^c^	189 (13.7)	151 (13.7)	30 (15.9)	8 (9.1)
Mixed & Other^d^	81 (5.9)	63 (5.7)	14 (7.4)	4 (4.6)
IMD quintile				
1	75 (5.4)	57 (5.2)	12 (6.4)	6 (6.8)
2	114 (8.3)	88 (8.0)	13 (6.9)	13 (14.8)
3	288 (20.9)	237 (21.5)	36 (19.1)	15 (17.1)
4	340 (24.6)	273 (24.8)	43 (22.8)	24 (27.3)
5	563 (40.8)	448 (40.6)	85 (45.0)	30 (34.1)
uCCI				
Low	1334 (97.4)	1074 (97.4)	183 (96.8)	87 (98.9)
High	36 (2.6)	29 (2.6)	6 (3.2)	1 (1.1)
SEWS category				
Low	880 (63.8)	721 (65.4)	105 (55.6)	54 (61.4)
Mid	307 (22.3)	233 (21.1)	53 (28.0)	21 (23.9)
Critical	193 (14.0)	149 (13.5)	31 (16.4)	13 (14.8)

a Includes White British, Irish and any other White background.

b Includes Black and Black British – African, Caribbean and any other Black background.

c Includes Asian and Asian British – Bangladeshi, Indian, Pakistani and any other Asian background.

d Includes Mixed – White and Black African, White and Black Caribbean, White and Asian, any other mixed background, Chinese, not stated and any other ethnic group.

A total of 1669 individual antibiotic prescriptions were administered to the cohort during the study period. In total, 1106 (80.1%) patients were treated with a single antibiotic, 259 (18.8%) with two and 15 (1.1%) with three antibiotics. Among patients treated with more than one antibiotic, there were only 31 prescriptions for aminoglycosides (27 gentamicin, 4 amikacin), with the majority (79.0%) being for betalactams in combination. Of all prescriptions, 1512 (90.6%) were intravenous and 157 (9.4%) were oral. Of oral antibiotics, 81 (51.6%) were for ciprofloxacin, 52 (33.1%) for co-amoxiclav, 17 (10.8%) for amoxicillin and 7 (4.5%) for cefalexin (data not shown).

In total, 1178 (85.4%) patients were treated with a concordant empirical antibiotic, ranging from 87.8% for KPB, 86.0% for ECB and 72.7% for PsAB. The most commonly prescribed empirical antibiotic was piperacillin/tazobactam (53.9% of prescriptions), followed by meropenem (22.6%) and co-amoxiclav (11.8%). Considered individually, the antibiotic most likely to be discordant was co-amoxiclav (22.8%, 45/197 prescriptions). The most commonly prescribed antibiotics are shown in the context of QEHB treatment guidelines in Table 2.

**Table 2.** Frequency of discordant empirical treatment by antibiotic as per QEHB guidelines

Antibiotic	Proportion discordant % (*N* total prescriptions)	Example indications QEHB guidelines
Meropenem	1.6 (377)	Severe sepsis associated with biliary/intra-abdominal or UTI, or of unknown cause^a^
Amikacin	0.0 (5)	Severe sepsis associated with biliary/intra-abdominal or UTI, or of unknown cause (penicillin allergy)^a^
Piperacillin/tazobactam	14.2 (899)	Acute cholangitis/cholecystitis/diverticulitis/peritonitis/intra-abdominal sepsis/complicated UTI/pyelonephritis/UTI in catheterised patient
Ciprofloxacin	15.5 (123)	Acute cholangitis/cholecystitis/diverticulitis (penicillin allergy)^b^, Peritonitis/intra-abdominal sepsis (penicillin allergy)^c^
Co-amoxiclav	22.8 (197)	Community acquired pneumonia, severe^d^

a In combination with vancomycin.

b In combination with metronidazole.

c In combination with metronidazole and gentamicin.

d In combination with clarithromycin.

### Factors predicting in-hospital death

Univariable and multivariable analyses were carried out for ECB and KPB only, as numbers for PsAB were small and whilst PsAB may have been community-onset, it was most likely to be healthcare-associated.

Multivariable analysis found that for ECB, discordant treatment was not associated with in-hospital death (Table 3). Non-urinary source ECB was associated with a threefold increase in odds of in-hospital death compared to the urinary source (adjusted OR 3.21, 95% CI 1.73–5.97, *P* < 0.001). Illness severity was associated with fivefold and 10-fold increased odds of in-hospital death for mid-level and critical SEWS, respectively (mid-level adjusted OR 6.37, 95% CI 3.13–12.97, *P* < 0.001; critical level adjusted OR 10.65, 95% CI 5.22–21.74, *P* < 0.001). For KPB, discordant treatment was associated with increased odds of in-hospital death on the univariable analysis (OR 4.78, 95% CI 1.25–18.33, *P* 0.012), but this effect was lost on the multivariable analysis (adjusted OR 4.03, 95% CI 0.96–16.86, *P* 0.06). There was no association between source of bacteraemia or SEWS score and in-hospital death for KPB.

**Table 3.** Multivariable analysis of risk factors for in-hospital death

		Univariable analysis		Multivariable analysis	
Patient characteristics	Death, *N*(%)	Death, OR (95% CI)	*P* value	Death, Adj OR (95% CI)	*P* value
All organisms					
Age (continuous)^a^		1.01 (1.00–1.02)	0.12	1.02 (1.00–1.03)	0.038
Male gender	39 (6.5)	1.00		1.00	
Female gender	32 (4.6)	0.70 (0.43–1.14)	0.15	0.80 (0.48–1.32)	0.38
uCCI low	68 (5.4)	1.00			
uCCI high	3 (8.6)	1.64 (0.49–5.49)	0.42		
Concordant treatment	60 (5.4)	1.00		1.00	
Discordant treatment	11 (6.2)	1.16 (0.60–2.25)	0.67	1.46 (0.73–2.92)	0.29
Urinary source	17 (2.7)	1.00		1.00	
Non-urinary source	54 (8.2)	3.23 (1.85–5.67)	<0.001	3.03 (1.71–5.38)	<0.001
SEWS low	17 (2.1)	1.00		1.00	
SEWS mid	29 (10.1)	5.37 (2.87–10.03)	<0.001	5.76 (3.08–10.78)	<0.001
SEWS critical	25 (13.9)	7.68 (3.98–14.81)	<0.001	7.85 (4.10–15.04)	<0.001
ECB					
Age (continuous)^a^		1.01 (1.00–1.03)	0.16	1.02 (1.00–1.03)	0.08
Male gender	31 (6.3)	1.00		1.00	
Female gender	29 (4.7)	0.73 (0.43–1.23)	0.24	0.85 (0.49–1.48)	0.57
uCCI low	57 (5.3)	1.00			
uCCI high	3 (10.3)	2.05 (0.60–7.01)	0.24		
Concordant treatment	53 (5.6)	1.00		1.00	
Discordant treatment	7 (4.5)	0.80 (0.36–1.79)	0.58	1.10 (0.47–2.58)	0.83
Urinary source	15 (2.7)	1.00		1.00	
Non-urinary source	45 (8.4)	3.37 (1.85–6.16)	<0.001	3.21 (1.73–5.97)	<0.001
SEWS low	13 (1.8)	1.00		1.00	
SEWS mid	23 (9.9)	5.96 (2.93–12.13)	<0.001	6.37 (3.13–12.97)	<0.001
SEWS critical	24 (16.1)	10.46 (5.05–21.66)	<0.001	10.65 (5.22–21.74)	<0.001
KPB					
Age (continuous)^a^		1.01 (0.98–1.05)	0.47	1.03 (0.98–1.07)	0.25
Male gender	8 (7.1)	1.00			
Female gender	3 (4.0)	0.54 (0.14–2.12)	0.37	0.61 (0.15–2.53)	0.50
uCCI low	11 (6.0)				
uCCI high	0 (0.0)	No deaths in KP			
Concordant treatment	7 (4.2)	1.00		1.00	
Discordant treatment	4 (17.4)	4.78 (1.25–18.33)	0.012	4.03 (0.96–16.86)	0.06
Urinary source	2 (3.0)	1.00		1.00	
Non-urinary source	9 (7.3)	2.53 (0.52–12.17)	0.23	2.76 (0.56–13.73)	0.22
SEWS low	4 (3.8)	1.00		1.00	
SEWS mid	6 (11.3)	3.22 (0.85–12.19)	0.07	2.74 (0.67–11.16)	0.16
SEWS critical	1 (3.2)	0.84 (0.09–7.88)	0.88	0.80 (0.08–7.63)	0.85

a Odds ratio is an approximation to the odds ratio for a one unit increase in age.

### Factors predicting ICU admission

Multivariable analysis found that for ECB, there was no association between discordant treatment and ICU admission (Table 4). The odds of ICU admission was increased with non-urinary *vs.* urinary source ECB (adjusted OR 1.99, 95% CI 1.08–3.65, *P* 0.026) and increased illness severity (mid-level SEWS adjusted OR 2.38, 95% CI 1.11–5.12, *P* < 0.026; critical SEWS adjusted OR 11.33, 95% CI 5.79–22.17, *P* < 0.001). For KPB, there was no association between discordant treatment or non-urinary source and ICU admission, but increased illness severity was again associated with increased odds of ICU admission (mid-level SEWS adjusted OR 3.69, 95% CI 1.01–13.56, *P* 0.049; critical SEWS adjusted OR 7.22, 95% CI 1.93–27.02, *P* 0.003).

**Table 4.** Multivariable analysis of risk factors for ICU admission

		Univariable analysis		Multivariable analysis	
Patient characteristics	ICU, N(%)	ICU, OR (95% CI)	*P* value	ICU, Adj OR(95% CI)	*P* value
All organisms					
Age (continuous)^a^		0.97 (0.96–0.98)	<0.001	0.97 (0.96–0.99)	<0.001
Male gender	42(7.0)	1.00		1.00	
Female gender	33 (4.8)	0.67 (0.42–1.07)	0.09	0.68 (0.41–1.12)	0.13
uCCI low	71 (5.7)	1.00			
uCCI high	4 (11.4)	2.16 (0.74–6.28)	0.15		
Concordant treatment	68 (6.1)	1.00		1.00	
Discordant treatment	7 (3.9)	0.63 (0.28–1.39)	0.25	0.80 (0.35–1.83)	0.59
Urinary source	25 (4.0)	1.00		1.00	
Non-urinary source	50 (7.6)	2.00 (1.22–3.27)	0.005	1.74 (1.03–2.93)	0.040
SEWS low	19 (2.3)	1.00		1.00	
SEWS mid	21 (7.3)	3.37 (1.77–6.39)	<0.001	2.75 (1.44–5.27)	0.002
SEWS critical	35 (19.4)	10.25 (5.56–18.92)	<0.001	10.25 (5.65–18.61)	<0.001
ECB					
Age (continuous)^a^		0.97 (0.96–0.99)	<0.001	0.97 (0.96–0.99)	<0.001
Male gender	32 (6.5)	1.00		1.00	
Female gender	25 (4.1)	0.61 (0.35–1.04)	0.07	0.62 (0.35–1.11)	0.11
uCCI low	54 (5.0)	1.00			
uCCI high	3 (10.3)	2.18 (0.64–7.44)	0.20		
Concordant treatment	52 (5.5)	1.00		1.00	
Discordant treatment	5 (3.2)	0.57 (0.23–1.46)	0.24	0.80 (0.30–2.14)	0.65
Urinary source	19 (3.4)	1.00		1.00	
Non-urinary source	38 (7.1)	2.20(1.25–3.88)	0.005	1.99 (1.08–3.65)	0.026
SEWS low	15 (2.1)	1.00		1.00	
SEWS mid	14 (6.0)	3.01 (1.42–6.36)	0.002	2.38 (1.11–5.12)	0.026
SEWS critical	28 (18.8)	10.89 (5.48–21.63)	<0.001	11.33 (5.79–22.17)	<0.001
KPB					
Age (continuous)^a^		0.98 (0.95–1.01)	0.12	0.98 (0.96–1.01)	0.20
Male gender	10 (8.9)	1.00		1.00	
Female gender	8 (10.5)	1.21 (0.45–3.23)	0.70	1.16 (0.41–3.25)	0.78
uCCI low	17 (9.3)	1.00			
uCCI high	1 (16.7)	1.95 (0.21–17.84)	0.55		
Concordant treatment	16 (9.6)	1.00		1.00	
Discordant treatment	2 (8.7)	0.89 (0.19–4.18)	0.89	0.75 (0.15–3.78)	0.73
Urinary source	6 (9.1)	1.00		1.00	
Non-urinary source	12 (9.8)	1.08 (0.39–3.03)	0.88	0.86 (0.29–2.53)	0.78
SEWS low	4 (3.8)	1.00		1.00	
SEWS mid	7 (13.2)	3.84 (1.05–14.11)	0.03	3.69 (1.01–13.56)	0.049
SEWS critical	7 (22.6)	7.36 (1.87–28.98)	<0.001	7.22 (1.93–27.02)	0.003

a Odds ratio is an approximation to the odds ratio for a one unit increase in age.

## Discussion

For ECB, increased illness severity and non-urinary source were associated with an increased odds of in-hospital death and ICU admission. For KPB, only increased illness severity was associated with an increased odds of ICU admission. We found no evidence in the multivariable analysis that discordant empirical antibiotic treatment was associated with either adverse outcome for either organism. However, confidence intervals for KPB were wide and an effect for KBP may have been missed.

Our findings are supported by a multi-centre prospective evaluation of empiric antibiotic treatment and outcome in GNB in 10 English hospitals (679 adult patients). They found that discordant empirical antibiotic treatment was not associated with all-cause mortality at 7 (adjusted OR 0.82, 95% CI 0.35–1.94, *P* 0.66) or 30 days (adjusted OR 0.92, 95% CI 0.50–1.66, *P* 0.77) [8] and that illness severity was an independent predictor of mortality. However, outcomes were not reported by the causative organism. A retrospective cohort study of 213 ECB and 203 KPB episodes from a tertiary hospital in the USA also found no association between discordant empiric antimicrobial treatment and in-hospital mortality for both organisms combined (HR 1.03, 95% CI 0.60–1.78) or each organism separately [10].

By contrast, a study of 1640 patients with community-onset bacteraemia (all organisms) admitted to a tertiary hospital in Spain found increased odds of death with discordant empirical antibiotic treatment (OR 2.0, 95% CI 1.22–3.33, *P* 0.006) [6]. This study also did not report outcomes by the causative organism. A subsequent study by this group analysed 4758 ECB episodes at a tertiary hospital in Spain and found the discordant empirical antibiotic treatment to be associated with an increased odds of 30-day mortality (OR 4.83, 95% CI 3.48–6.71, *P* < 0.001) [9]. This study period was over a decade ago (data collected between 1991 and 2007) and in a setting with higher rates of antibiotic resistance than the UK [22]. The reported fluoroquinolone resistance was 27% of isolates, compared to 18.9% ciprofloxacin resistance in our study. Additionally, 29% of ECB in this study were nosocomial. Similar to our study, however, they found an increased mortality rate in non-urinary source bacteraemias (pneumonia and intra-abdominal infection). A number of other studies that have shown a detrimental effect with discordant empirical antibiotic were largely carried out in patients with severe sepsis and septic shock, who may be less representative of patients with bacteraemia [7, 23, 24].

The limitations of our study include the small numbers of cases for KPB and it is possible that this hampered our ability to detect an association between discordant treatment and in-hospital mortality on multivariable analysis despite finding one on the univariable analysis (adjusted OR 4.03, 95% CI 0.96–16.86, *P* 0.06). The recently introduced mandatory surveillance of KPB may give insight into the incidence and trends of antibiotic resistance and provide data for larger studies that may inform treatment guidelines. Additionally, we had no data on events preceding admission, including antibiotic prescribing and were therefore unable to identify which admissions were healthcare-associated. Our identification of patients with urinary source used ICD-10 codes and positive urine cultures within −30 to +2 days of admission. We may therefore have misclassified patients as having a non-urinary source if they did not have a urine culture sent, or had cultures sent outside this timeframe, or were simply negative on culture. This was a single-site study and the results may not be applicable to other settings.

A major strength of our study is that we had a large high-quality dataset (>1300 patients with GNB) including patient-level data on demographics, admissions, prescriptions and microbiology including resistance data. We were, therefore, able to examine the relationship between clinical outcomes and treatment concordance by the organism, adjusting for the effects age, sex, illness severity and infection source. Our study adds to the literature on clinical outcomes in patients with GNB in the context of rising incidence and antibiotic resistance rates.

We defined concordance of empirical treatment based on antibiotics prescribed ±1 day from admission, in keeping with other studies. An explanation for our findings may be that patients categorised as ‘discordant’ were subsequently treated with concordant antibiotics. Alternatively, in-vitro resistance may not necessarily correlate with clinical outcomes. As there is some evidence that oral treatment may be equivalent to the intravenous route for severe infections, a sensitivity analysis was undertaken where all antibiotics (including oral antibiotics other than ciprofloxacin) were classified as concordant or discordant based on in vitro susceptibility alone and this analysis did not change the results.

Our study highlights a number of issues around prescribing for GNB. For ECB (which accounted for 79.9% of patients in our study), illness severity and non-urinary source were associated with increased odds of both in-hospital death and ICU admission. This highlights the importance of prompt clinical assessment and management of adverse physiology and the potential use of these parameters, which are assessed at admission, to guide empirical prescribing decisions. As discordant treatment was not associated with adverse outcomes, our results suggest that in the context of increasing rates of resistance, rather than broadening the spectrum of empirical antibiotics for all patients with suspected Gram-negative bacteraemia, use of narrow-spectrum agents with the tailoring of antibiotic therapy – once microbiological data are available – may be a safe approach to take in patients with a urinary source and without signs of severe illness. Future research should aim to investigate further the relationship between patient outcomes, clinical and demographic factors, infection focus, causative organism and resistance profile, particularly for KPB, and explore how this information can be used to risk stratify patients and optimise antibiotic use.

## Data Availability

The data that support the findings of this study are available from MJG. Restrictions apply to the availability of these data, which were used under licence for this study. Data are available from the authors with the permission of MJG.

## Supplementary material

For supplementary material accompanying this paper visit http://dx.doi.org/10.1017/S0950268820002083. click here to view supplementary material
