# Supplementary material for: The relationship between clinical outcomes and empirical antibiotic therapy in patients with community-onset Gram-negative bloodstream infections: a cohort study from a large teaching hospital
Source: Epidemiol Infect. 2020 Sep 11;148:e225. doi: 10.1017/S0950268820002083 (PMC7556992; doi:10.1017/S0950268820002083)
Supplement: Supplementary file 1 [file S0950268820002083sup001.docx]

*Epidemiology & Infection*

The relationship between clinical outcomes and empirical antibiotic therapy in patients with community-onset Gram-negative bloodstream infections: a cohort study from a large teaching hospital

A. Aryee, P. Rockenschaub, M. J. Gill, A. Hayward, L. Shallcross

**Supplementary material**

1. **List of ICD codes used to identify urinary source bacteraemia**

| N10 | Acute tubulo-interstitial nephritis |
| --- | --- |
| N11.0-9 | Chronic tubulo-interstitial nephritis |
| N12 | Tubulo-interstitial nephritis, not specified as acute or chronic |
| N13.6 | Pyonephrosis |
| N15.1 | Renal and perinephric abscess |
| N30.0-9 | Cystitis |
| N34.0 | Urethral abscess |
| N39.0 | Urinary tract infection, site not specified |
| N41.0-9 | Inflammatory diseases of prostate |

1. **Identification of urinary source bacteraemia**

Primary or secondary ICD code indicating urinary infection

N = 557

Entire cohort

N = 1380

Positive urine culture where organism matches that on blood culture

N = 330

N = 235
